# Japanese mothers’ utterances about agents and actions during joint picture-book reading

**DOI:** 10.3389/fpsyg.2014.00357

**Published:** 2014-05-08

**Authors:** Toshiki Murase

**Affiliations:** Faculty of Law and Literature, Shimane UniversityMatsue, Japan

**Keywords:** scaffolding, referential choice, information seeking, elaboration, mother–child communication, toddlers, picture-book reading, Japanese

## Abstract

This study extended the research on the scaffolding provided by mothers while reading picture books with their children from a focus on conversational styles related to labeling to a focus on those related to agents and actions to clarify the process by which language develops from the one-word to the syntactic stage. We clarified whether mothers decreased the degree of scaffolding in their initiation of conversations, in the responses to their children’s utterances, and in the choice of referential ranges of their utterances. We also investigated whether maternal conversational styles contributed to the development of their children’s vocabularies. Eighteen pairs of Japanese mothers and their children were longitudinally observed when the children were 20 and 27 months of age. The pairs were given a picture book depicting 24 animals engaged in everyday behavior. The mothers shifted their approach in the initiation of conversation from providing to requesting information as a function of their children’s age. The proportion of maternal elaborative information-seeking responses was positively correlated with the size of their children’s productive vocabulary. In terms of referential choices, mothers broadened the range of their references as their children aged. In terms of the contribution of maternal conversational styles to children’s vocabulary development, the use of a maternal elaborative information-seeking style when the children were 20 months of age predicted the size of the children’s productive vocabulary at 27 months. These results indicate that mothers decrease the degree of scaffolding by introducing more complex information into the conversations and transferring the role of actively producing information to their children by requesting information as their children develop. The results also indicate that these conversational styles promote the development of children’s vocabularies during the transition from the one-word to the syntactic stage.

## INTRODUCTION

During the second year of life, children experience remarkable linguistic development, rapidly increasing their vocabulary and beginning to produce word combinations. The role of hearing others’ language in this development has been investigated, and picture-book reading is among the activities that have been suggested to promote children’s language development (Fletcher and Reese, 2005). For example, Whitehurst and colleagues demonstrated the effects of dialogic reading on vocabulary development (Arnold and Whitehurst, 1994).

Speech by caregivers during joint picture-book reading has been shown to support children’s language development. In this context, caregivers demonstrated greater lexical diversity, syntactical complexity, and more frequent topic-continuing responses (Hoff-Ginsberg, 1991) and focused more on labeling than they did in other situations (Tardif et al., 1999; Choi, 2000). Additionally, conversations between caregivers and children were characterized by attempts to attract the attention of the other party, requests for information, labeling, and feedback (Ninio and Bruner, 1978; DeLoache and DeMendoza, 1987).

Most studies of conversations between caregivers and toddlers have used picture books dominated by pictures of individual items (DeLoache and DeMendoza, 1987; Tardif et al., 1999; Murase et al., 2005; Chan et al., 2009) and have focused on conversations about object names. To date, few studies have analyzed more complex conversations between caregivers and toddlers that move beyond labeling. The bias favoring conversations about object names rests on the dominance of object names, especially among English-speaking children, during their first stage of vocabulary acquisition. However, they show a slow linear increase in their use of verbs and other predicates, with the greatest gains occurring when their vocabulary consists of 100–400 words (Bates et al., 1994). Japanese children share this tendency, although nouns are less predominant than among their English-speaking counterparts (Ogura, 1999). We need to extend our knowledge about mother–child conversations from a narrow focus on object names to a more expansive focus on utterances about the actions of agents. The present study used a picture book that included a series of scenes of an animal performing an action. We analyzed the utterances about agents and actions offered by caregivers in the service of clarifying the process by which language develops from the single-word to the syntactic stage.

Bruner and colleagues proposed that caregivers provide language assistance to their children and mentioned scaffolding as one of the strategies employed in this process. Scaffolding consists of the structure provided by caregivers for those aspects of the task at hand that are beyond children’s capacity (Wood et al., 1976; Bruner, 1981). They also identified raising the ante as another strategy in this domain. This strategy entails caregivers’ efforts to find ways of challenging children to incorporate a task they have already mastered into a more complex routine for achieving a more remote end. As children grow older, caregivers reduce the degree of scaffolding and transfer the role to the children themselves with the expectation that the children will be autonomous participants in conversations (Wood et al., 1976; Bruner, 1981).

Wood et al. (1976) identified six processes involved in scaffolding: recruitment, reduction in degrees of freedom, direction maintenance, marking critical features, frustration control, and demonstration. We focus on reduction in degrees of freedom, direction maintenance, and demonstration in the present study. Reducing the degrees of freedom entails systematically simplifying the task by reducing the number of constituent acts required to reach a solution. Maintaining direction means that caregivers keep children focused on the pursuit of a particular goal. Demonstration involves the idealization by caregivers of the task in question, including completing or expanding on an action executed by their children, with the expectation that the children will then imitate the new version.

We investigated the scaffolding provided by caregivers in conversations before and after the performance of conversational elements by children. Our first research question relates to the degree to which caregivers provide scaffolding at the beginning of a conversation, before children produce utterances about agents and actions. From the perspective of scaffolding, caregivers’ requests for information provide less scaffolding than does their provision of information. Requests for information provide children with a cue but not concrete information; this stands in contrast to the practice of providing concrete information in the form of a full model. In this regard, requesting information can be seen as requiring that the children control more degrees of freedom compared with providing information. Previous research has shown that as children increased in age, caregivers increased the frequency with which they initiated conversational episodes with requests for information (DeLoache and DeMendoza, 1987; Murase et al., 1998) and increased the frequency with which they requested information in general (Bornstein et al., 1992; Fernald and Morikawa, 1993). Unlike previous studies that investigated all requests for information, we differentiated caregivers’ requests for information about agents from those for information about actions, and investigated whether caregivers initiated a larger proportion of both types of conversations by requesting information and a smaller proportion by providing information as a function of the increase in the children’s age.

Our second research question related to the degree to which caregivers provided scaffolding in their responses after children provided information about agents and actions. Elaboration, which involves adding new information, can be seen as providing less scaffolding and raising the ante because it extends the children’s utterances with the expectation that they will incorporate the new information into a more complex conversation. Previous studies have found that caregivers raise the ante in response to children’s utterances as a function of children’s age or linguistic ability and that maternal elaboration was more strongly related to children’s linguistic ability than to their age (Ninio and Bruner, 1978; Murase et al., 2005). Thus, we hypothesized that the proportion of elaborative responses by caregivers would increase as children grew older.

In contrast, other-repetition involves repeating information provided by the other party in a dyadic interaction. Although other-repetition is used for various instructional and communication purposes, such as correction, acknowledging receipt of information, asking for clarification, and asking for confirmation (Demetras et al., 1986; Otomo, 2001; Huang, 2011), other-repetition can be interpreted as providing scaffolding. First, maternal other-repetition can serve as demonstration in the scaffolding process. Mothers may imitate children’s attempts at information giving in an idealized form with the expectation that children will then imitate them. Second, maternal other-repetition can maintain the trajectory of the process. Mothers have the role of keeping younger children focused on the pursuit of a particular goal. In conversations, mothers may encourage children to continue a coherent dialog about a theme by explicitly repeating their children’s utterances. Thus, we hypothesized that the proportion of other-repetition responses by caregivers would decrease as children grew older.

Additionally, we also investigated the extent of the information introduced by caregivers into conversations, the third aspect of scaffolding, which involves the issue of referential choice. Setting a limit on referential choices can act as a kind of scaffolding because caregivers reduce the degrees of freedom available to their children when referring to the scene.

Individuals in different cultures attend to different aspects of the same scene. Indeed, cross-cultural studies have found that East Asians are more attentive than are North Americans to contextual and relational information and that North Americans are more attentive than are East Asians to individual objects (Masuda and Nisbett, 2001; Masuda et al., 2008; Kuwabara et al., 2011; Kuwabara and Smith, 2012). Cross-linguistic studies of vocabulary development have revealed that Mandarin- and Korean-speaking children are less biased in favor of nouns than are English-speaking children and that Mandarin- and Korean-speaking caregivers use fewer nouns and more verbs in speaking to their children than do their English-speaking counterparts (Choi and Gopnik, 1995; Tardif, 1996; Tardif et al., 1997; Kim et al., 2000). A few studies have suggested that Japanese-speaking children are also less biased in favor of nouns than are English-speaking children (Ogura, 1999; Ogura et al., 2006). The results of these studies indicate that individuals choose the targets of their attention in a given scene, for example, an agent or action, in culturally specific ways.

Japanese is a pro-drop language, which allows the optional omission of either agent or object arguments (Guerriero et al., 2006). For example Japanese allows, *Neko ga miruku o nondeiru* (A cat is drinking milk), *Miruku o nondeiru* (is drinking milk), *Neko ga nondeiru* (A cat is drinking), and *Nondeiru* (Drinking) for the expression of *A cat is drinking milk*. This means that Japanese speakers choose which elements to include in their description of a scene.

According to the foregoing discussion, Japanese caregivers make two kinds of choices about what to refer to in a scene: one involves the aspects to which they will attend, and the other involves the elements to which they will verbally refer. This study investigated how Japanese caregivers change their verbal referential choices in utterances directed at their children during joint picture-book reading. Previous studies about referential choice have focused on pragmatic explanations. For example, caregivers use more non-lexical than lexical forms in reference to given information (Guerriero et al., 2006), and they tend to mention unexpected more than usual objects (Sethuraman and Smith, 2010). However, changes in the range of references by caregivers have not been clarified.

Working from the perspectives of scaffolding and raising the ante, we hypothesized that caregivers would narrow their references to specific elements within a scene when communicating with younger children, whereas they would broaden this range as children got older. Specifically, we predicted that caregivers would refer only to the agents or the actions in a scene more frequently when communicating with younger than with older children. In contrast, we predicted that caregivers would refer to both the agents and the actions in a scene more frequently when communicating with older than with younger children.

As this study used a longitudinal design, our fourth research question addressed the effects of maternal conversational style on children’s vocabulary development. Previous studies have found that maternal responsiveness (Tamis-LeMonda et al., 2001; Tamis-LeMonda and Bornstein, 2002) and maternal lexical richness (Bornstein et al., 1998; Hoff and Naigles, 2002) had positive effects on children’s vocabulary development. The present study investigated the effects of the styles used by mothers to initiate conversations about agents and actions, their approaches to responding to children’s information-giving utterances about agents and actions, and the effects of their referential choices about information about agents and actions on their children’s vocabulary development.

In summary, this study investigated four issues. First, we clarified the degree of scaffolding provided by caregivers when initiating conversations with their children by providing or requesting information about agents and actions. We hypothesized that the proportion of conversations initiated by caregivers about both agents and actions by providing information would decrease and the proportion initiated by seeking information would increase as the children’s age increased (hypothesis 1). Second, we determined the degree of scaffolding provided by caregivers in their responses to their children’s provision of information about agents and actions. We hypothesized that in response to information provided by their children caregivers would engage in more elaboration and less other-repetition as the age of their children increased (hypothesis 2). Third, we clarified changes in caregivers’ referential choices. We hypothesized that caregivers reduce references to agents or actions alone and increase references to both agents and actions as children age (hypothesis 3). Fourth, we investigated the effects of maternal conversational styles at an earlier time on the size of children’s productive vocabulary at a later time, controlling for children’s productive vocabulary size at the earlier time.

## MATERIALS AND METHODS

### PARTICIPANTS

Eighteen pairs of Japanese mothers and their children (8 boys and 10 girls) were observed when the children were 20 (range: 20.0–20.13, mean: 20.05) and 27 (range: 26.29–27.17, mean: 27.08) months of age. Based on the norms of the Japanese MacArthur Communicative Development Inventory (JCDI), which show that the median size of the productive vocabulary size of 20-month-old boys is 63 and that that of 20-month-old girls is 95, we studied children of this age because they have a substantial productive vocabulary (Watamaki and Ogura, 2004). Moreover, Japanese children exhibit a growth spurt in the size of their vocabulary at an average of 20 months of age (Kobayashi et al., 2013). We considered 27-month-old children to be in the syntactic stage based on data about maximum sentence length and the age at which they begin to combine words. According to JCDI norms (Watamaki and Ogura, 2004), more than 90% of 27-month-old Japanese children had started to combine words, and the median maximum sentence length was 4.27 words among 27-month-old boys and 5.78 words among 27-month-old girls. Seven children were first born, seven were second born, and four were born third or later in their families. Fourteen children were cared for by their mothers at home, and four children were cared for at day nurseries during the day.

### PROCEDURE

Each mother–child pair visited the playroom of the university when the child was 20 and 27 months of age. The book-reading session lasted 7 min to enable the young children to maintain their attention on the book. The experimenter left the room during the reading and then returned and stopped the session after 7 min even if the participants had not finished looking at all the pictures to minimize the children’s frustration. As this study is a part of a project examining mother–child interaction, a 7-min play session simulating a dressing situation preceded and another 7-min play session simulating a cooking situation followed the book-reading session. The mothers also completed questionnaires regarding their children’s productive vocabulary. When their children were 20 months of age, these questionnaires addressed whether and how their children produced a word for 82 familiar items and whether and how their children produced a word in 62 situations. The 82 familiar items included animals (e.g., dog), foods and beverages (e.g., banana), clothing (e.g., shirt), small household items (e.g., spoon), and places (e.g., bathroom). The 62 situations included interactions (e.g., giving something to a family member), requests (e.g., asking to be picked up), routine events (e.g., getting up in the morning), experiences (e.g., eating a favorite food), and actions (e.g., sitting on a chair). When their children were 27 months of age, the questionnaires asked the questions about 112 familiar items and 76 situations. The 112 familiar items included the 82 used at 20 months as well as several additional items (e.g., pigeon, pineapple). The 76 situations included the 62 used at 20 months as well as several additional situations (e.g., asking about why certain actions were taken and correcting the verbal expressions of others). These instruments were used to assess the size of the children’s productive vocabulary. We assessed children’s productive vocabulary size by counting the different word types reported by mothers in response to both familiar items and situations.

### MATERIALS AND CODING

#### Materials

**FIGURE 1:**
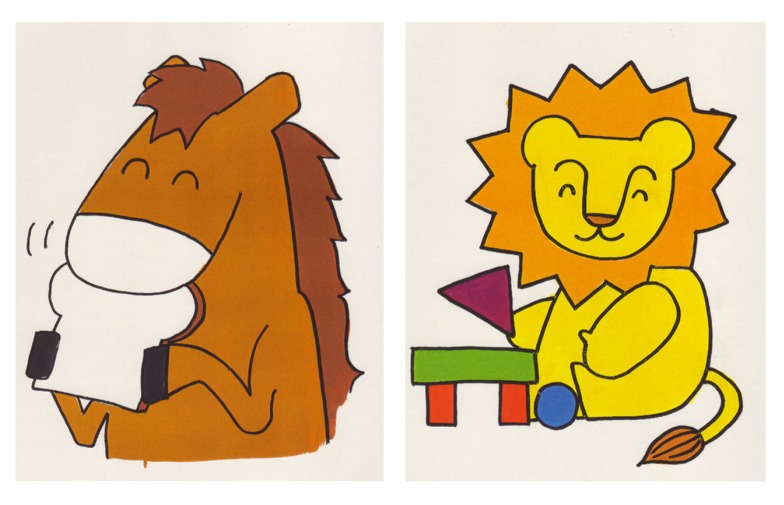
**Examples of pictures used**.

Each pair received a wordless picture book created for this research that depicted 24 animals engaged in ordinary behaviors (e.g., a horse eating bread, a lion playing with blocks; **Figure 1**).

#### Utterances for analysis

We analyzed utterances during episodes in which the mother and child jointly looked at the picture book. We defined episodes in reference to the reading cycle (Ninio and Bruner, 1978). The onset of an episode was recorded when (a) the book was open to a picture within 50 cm from both the child and mother and (b) the child or mother was pointing, gesturing, or vocalizing in some fashion that was directed to the content of the book. The offset of an episode was recorded when any of the following occurred: (a) the book was closed, (b) a new picture was introduced, or (c) either the child’s or the mother’s attention was withdrawn from the picture for more than 5 s. An utterance was defined as an informational unit that conveyed an idea. We divided speech into utterances based on syntactical information and supplemented this with observations of pauses and prosodic information when necessary to identify the endpoints of utterances.

#### Coding of individual utterances by mothers

Utterances including the names of animals appearing in a picture in the book were coded as instances of giving information about agents. We included not only conventional naming using nouns but also conventional onomatopoeias in this category because onomatopoeias are often used as labels for animals during conversations between Japanese mothers and children (e.g., *wanwan* [woof-woof] and *inu* [dog] were both coded as giving information about agents). Utterances with information about the actions performed by the animals in a picture were coded as giving information about actions. We included not only conventional verbs but also conventional onomatopoeias in this category because onomatopoeias are also often used to express actions in conversations between Japanese mothers and children (e.g., both *gokungokun* [onomatopoeia for drinking] and *nonderu* [drinking] were coded as giving information about actions).

Utterances requesting the names of animals appearing in a picture in the book were coded as seeking information about agents (e.g., *Kore dare?* [Who is he?]). Utterances requesting information about actions depicted in a picture were coded as seeking information about actions (e.g., *Nani shiteru?* [What is he doing?]). Ambiguous utterances requesting information (e.g., *Kore wa?* [And this?]) were excluded from both categories.

The above categories were multiply coded. For example, statements by mothers such as *An elephant is washing his body* were coded as providing information about agents and actions, and utterances such as *What is the lion doing?* were coded as requests for information about actions and providing information about agents.

#### Coding of individual utterances by children

Utterances in which children provided information about agents and actions were coded using the same criteria as those used for mothers. Information requests issued by children were not differentiated according to whether their focus was on agents or on actions.

#### Coding of conversation initiation

The style of initiating conversations about agents and actions in response to each picture was coded in terms of three categories: maternal initiation by information giving, maternal initiation by information seeking, and children’s initiation. In terms of information about agents, maternal initiation by information giving was coded when mothers initiated a conversation about agents with information giving preceding information seeking (e.g., mother said, *“He is a dog*,” and then child said*,* “*Dog*”). Maternal initiation by information seeking was coded when mothers initiated a conversation about agents with information seeking preceding information giving (e.g., mother said, “*Who is he? He is a dog*,” and then the child said, “*Dog*.”). Children’s initiation was coded when children initiated a conversation about agents by information giving or information seeking (e.g., the child said, “*Dog*,” and then mother said, “*Yes, he is a dog*”). We coded data on the initiation of conversations about actions in the same way as we coded data on the initiation of conversations about agents.

#### Coding of mothers’ responses to children’s provision of information

Mothers’ utterances in the first and second turns following utterances in which children provided information about agents and actions were coded as “elaborative information giving,” “elaborative information seeking,” or “other-repetition.” We defined elaboration as adding new information during conversations. Recent studies have shown that different styles of elaboration should be differentiated from one another (Fivush et al., 2006; Melzi et al., 2011), and we distinguished two types of elaboration: giving and seeking elaborative information. Both types can be viewed as ways to raise the ante as they introduce more complex information into conversations. However, requesting elaborative information provides less scaffolding than does offering elaborative information because the former involves questions rather than the provision of concrete information. Elaborative information giving was defined as the provision of information that had not been produced by the children during the episode in question. For example, a code of elaborative information giving was given when a mother said, “*He is washing his body*” in response to her child’s saying, “*Elephant*,” but this code was not given when a mother responded to the same utterance with, “*He is an elephant*.” Elaborative information seeking was defined as a request for information that had not been previously produced by the children during the episode in question. For example, a code of elaborative information seeking was given when a mother asked, “*What is he doing?*” following a child’s saying, “*Elephant*.” Other-repetition was defined as information provided by mothers that repeated the information provided by children in the last turn. For example, a code of other-repetition was given when a mother said, “*He is a lion*” after her child said, “*Lion*,” but this code was not given when a mother said, “*He is an animal*” following her child’s utterance of “*Lion.*” These three categories were multiply coded. For example, codes of other-repetition and elaborative information giving were given when a mother said, “*The elephant is washing his body*” after her child said, “*Elephant*” because the utterance both repeated information provided by the child and offered new information.

#### Inter-coder reliability

Two coders independently coded all utterances. The *kappa* values representing the reliability of maternal information giving about agents and actions and maternal requests for information about agents and actions were 0.98, 0.89, 0.91, and 0.95, respectively. The *kappa* values for children’s information giving about agents and actions and for children’s requests for information were 0.97, 0.87, and 0.84, respectively. Final coding was determined by discussions between the two coders.

### ASSESSMENT OF CHILDREN’S PRODUCTIVE VOCABULARY

We assessed the children’s productive vocabulary at 20 and 27 months of age according to two criteria. The first criterion was the number of different word types produced by the children during a 7-min book-reading session and two 7-min play sessions. The second criterion was the number of different word types reported by mothers on the questionnaires. The two criteria were strongly correlated [*r*(16) = 0.80 at 20 months, *r*(16) = 0.83 at 27 months], and children showed stability in their word productivity. The productive vocabulary sizes estimated via observations of the 21-min sessions conducted at 20 and 27 months of age were strongly correlated [*r*(16) = 0.74], and the productive vocabulary sizes estimated by mothers at these two stages were also strongly correlated [*r*(16) = 0.88].

## RESULTS

The mean numbers of pictures referenced by mothers and children and the mean numbers of utterances by mothers and children per picture are presented in **Table 1**. A repeated-measures ANOVA on the number of pictures referenced by mothers and children revealed a main effect of children’s age. More pictures were referenced by mothers and children when the children were 27 months of age than when they were younger, *F*(1,17) = 8.35, *p* < 0.05, η^2^ = 0.33. The numbers of pictures referenced when children were 20 and 27 months were consistent [*r*(16) = 0.56, *p* <0.05].

**Table 1:** Mean numbers of pictures referenced and mean number of utterances per picture (SDs in parentheses).

	20 Months	27 Months
Number of pictures referenced by mothers and children	17.22 (5.12)	20.11 (2.74)
Number of utterances per picture by mothers	4.95 (2.51)	5.98 (1.96)
Number of utterances per picture by children	1.75 (1.62)	3.27 (1.07)

Repeated-measures ANOVAs on the number of utterances by mothers and by children per picture revealed a main effect of children’s age on children’s utterances, *F*(1,17) = 18.41, *p* <0.001, η^2^ = 0.84, and a marginal main effect of children’s age on maternal utterances, *F*(1,17) = 3.09, *p* < 0.10, η^2^ = 0.15. Both mothers and children increased the number of utterances per picture as children developed from 20 to 27 months. The number of maternal [*r*(16) = 0.41, *p* <0.10] and child [*r*(16) = 0.44, *p* <0.10) utterances per pictures were moderately consistent between 20 and 27 months of age.

### INITIATION OF CONVERSATIONS BETWEEN MOTHERS AND CHILDREN (HYPOTHESIS 1)

The following analysis examines the results of the repeated-measures ANCOVAs treating children’s age as an independent variable and children’s observed productive vocabulary size at 20 months as a covariate. We also treated the productive vocabulary size reported by mothers at 20 months as a covariate. When the second analysis produced essentially the same results as the first, we do not report the second analysis. When the second analysis produced results that differed from the first, we report the second results after the first.

The three ways of initiating conversations (maternal information giving, maternal information seeking, and children’s initiation) were analyzed separately for the conversation for both agents and actions. Comparisons were calculated by dividing the number of pictures in response to which mothers and children initiated a conversation about agents (actions) in each of these ways by the total number of pictures in response to which mothers and children referred to agents (actions). **Table 2** presents the mean proportions of the three styles of initiating conversations about agents and actions.

**Table 2:** Mean proportions of initiation types (SDs in parentheses).

	20 Months	27 Months
**Initiation of conversation about agents**
Maternal information giving	0.43 (0.27)	0.21 (0.21)
Maternal information seeking	0.52 (0.28)	0.58 (0.27)
Children’s initiation	0.05 (0.12)	0.20 (0.21)
**Initiation of conversation about actions**
Maternal information giving	0.78 (0.17)	0.43 (0.22)
Maternal information seeking	0.22 (0.16)	0.50 (0.20)
Children’s initiation	0.01 (0.03)	0.08 (0.12)

In terms of the initiation of conversations about agents, a series of ANCOVAs revealed main effects of children’s age on the proportion of utterances in which mothers provided information and children initiated conversations, *F*(1,16) = 7.38, *p* <0.05, η^2^ = 0.32; *F*(1,16) = 8.95, *p* < 0.01, η^2^ = 0.36, but no main effect of children’s age on the proportion of maternal information-seeking utterances was observed. As the children grew older, the mothers initiated a smaller proportion of the conversations by providing information, and the children initiated a larger proportion of the conversations.

In terms of the initiation of conversations about actions, a series of ANCOVAs showed main effects of children’s age on the proportion of maternal information-giving and maternal information-seeking utterances, *F*(1,16) = 16.76, *p* <0.001, η^2^ = 0.51; *F*(1,16) = 14.88, *p* < 0.001, η^2^ = 0.48, but no main effect of children’s age on the proportion of conversations initiated by children was observed. Mothers initiated a smaller proportion of conversations about actions by information giving and initiated a larger proportion by information seeking as their children aged from 20 to 27 months.

When we used the productive vocabulary size reported by mothers at 20 months as a covariate, the results of the ANCOVAs showed that the proportion of child-initiated utterances about agents did not significantly change as a function of their age.

### MATERNAL RESPONSES TO CHILDREN’S PROVISION OF INFORMATION (HYPOTHESIS 2)

#### Responses to children’s information-giving utterances about agents

**FIGURE 2:**
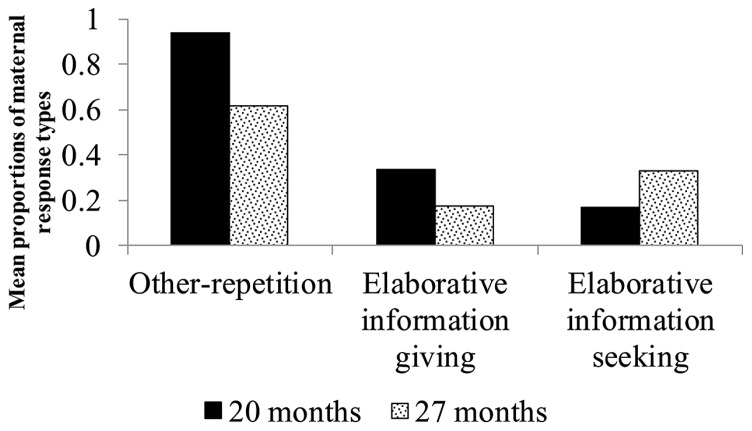
**Mean proportions of maternal response types to children’s information-giving utterances about agents**.

The two turns by mothers following their children’s first information-giving utterance about agents for each picture were analyzed. We selected only the first information-giving utterance about agents for each picture because the conversational sequence leading to later information-giving utterances made by the children was probably affected by the first such utterance regarding the picture. Data from only those mother–child pairs in which the child produced information about agents at both 20 and 27 months of age were included in this analysis. Fourteen pairs met this criterion (The mean number of episodes analyzed at 20 and 27 months were 6.1 and 12.3, respectively). Proportions were computed by dividing the number of pictures in response to which mothers produced elaborative information-giving, elaborative information-seeking, and other-repetition utterances following children’s information-giving utterances about agents by the number of pictures in response to which children produced information-giving utterances about agents. A series of ANCOVAs showed a main effect of children’s age on the proportion of maternal other-repetition utterances, *F*(1,12) = 25.20, *p* < 0.001, η^2^ = 0.68 (*M* = 0.94, SD = 0.09 for the proportion at 20 months; *M* = 0.62, SD = 0.23 for the proportion at 27 months;* M* = 5.43, SD = 4.94 for the number of pictures at 20 months; *M* = 8.50, SD = 4.03 for the number of pictures at 27 months), but no main effect of children’s age on the proportion of maternal elaborative information-giving (*M* = 0.34, SD = 0.27 for the proportion at 20 months; *M* = 0.17, SD = 0.15 for the proportion at 27 months;* M* = 2.29, SD = 2.81 for the number of pictures at 20 months; *M* = 2.43, SD = 2.28 for the number of pictures at 27 months) and elaborative information-seeking (*M* = 0.17, SD = 0.18 for the proportion at 20 months; *M* = 0.33, SD = 0.23 for the proportion at 27 months;* M* = 1.36, SD = 1.82 for the number of pictures at 20 months; *M* = 4.21, SD = 2.75 for the number of pictures at 27 months) utterances was observed. The proportion of maternal other-repetition responses to children’s information-giving utterances about agents decreased as the children aged from 20 to 27 months (**Figure 2**).

Murase et al. (2005) found that the proportion of maternal elaborative information-seeking utterances was more strongly correlated with children’s productive vocabulary size than with children’s age. Therefore, we analyzed the correlation between maternal elaborative information-seeking utterances and children’s productive vocabulary size at 20 and 27 months. A significant positive relationship was found between the proportion of maternal elaborative information-seeking utterances and children’s reported productive vocabulary size when children were 20 months of age [*r*(12) = 0.62, *p* < 0.05], but the relationship between the proportion of elaborative information-seeking utterances and children’s observed productive vocabulary size was weaker [*r*(12) = 0.38, n.s.]. Marginally significant positive relationships were found between the proportion of maternal elaborative information-seeking utterances and the size of both the reported [*r*(16) = 0.42, *p* < 0.10] and the observed [*r*(16) = 0.43, *p* < 0.10] productive vocabularies when children were 27 months of age. These results show moderately positive relationships between maternal elaborative information-seeking utterances and the size of children’s productive vocabulary.

#### Responses to children’s information-giving utterances about actions

**FIGURE 3:**
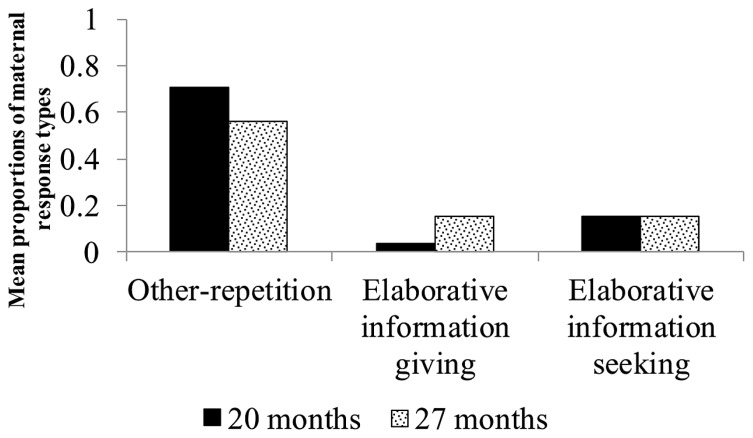
**Mean proportions of maternal response types to children’s information-giving utterances about actions**.

The two turns by mothers following their child’s first information-giving utterance about actions were analyzed for each picture. We selected only the first information-giving utterance about actions produced by the child for the same reason that we followed this strategy in our analysis of responses to children’s information-giving utterances about agents. Only those pairs in which the child offered information about actions at both 20 and 27 months of age were included in this analysis. Data from 10 pairs were used in this analysis (The mean numbers of episodes analyzed at 20 and 27 months were 1.8 and 5.8, respectively). Proportions were computed by dividing the number of pictures in response to which mothers produced elaborative information-giving, elaborative information-seeking, and other-repetition utterances following children’s information-giving utterances about actions by the number of pictures in response to which children produced information-giving utterances about actions. A series of ANCOVAs revealed no significant main effect of the proportions of the three dependent variables (**Figure 3**).

### RANGE OF REFERENCES IN RESPONSE TO PICTURES (HYPOTHESIS 3)

We categorized the range of references provided by mothers in response to each picture into four types: references to both agents and actions, references to agents only, references to actions only, and references to neither agents nor actions. We categorized the series of utterances in response to pictures as references to agents and actions when mothers provided at least one piece of information about both agents and actions. We categorized responses as references to agents only when mothers offered information about agents but not about actions, even if other information, such as arguments, was provided. We categorized responses as references to actions only when mothers offered information about actions but not about agents, even if other information was provided. We categorized responses as references to neither agents nor actions when mothers did not provide information about agents or actions.

**FIGURE 4:**
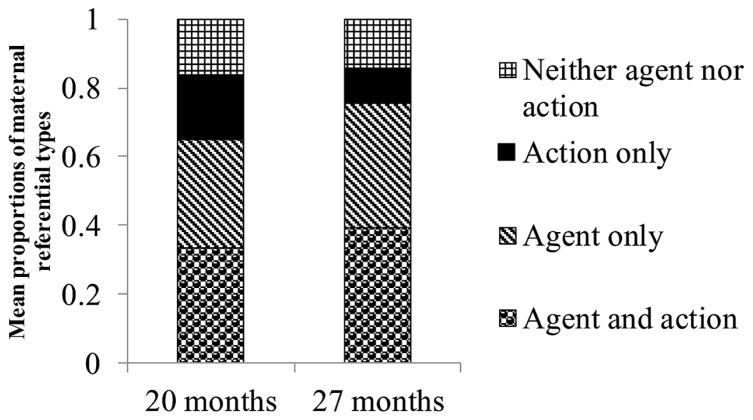
**Mean proportions of mothers’ referential types for information-giving utterances about a picture**.

A series of ANCOVAs on the proportions of the four categories (agents and actions, agents only, actions only, and neither agents nor actions) revealed a main effect of children’s age on maternal actions-only responses, *F*(1,16) = 9.29, *p* < 0.01, η^2^ = 0.37. The proportion of mothers’ actions-only responses decreased between 20 and 27 months of age (*M* = 0.19, SD = 0.14 at 20 months; *M* = 0.10, SD = 0.10 at 27 months). We found no main effects of children’s age on the other three responses (**Figure 4**).

**FIGURE 5:**
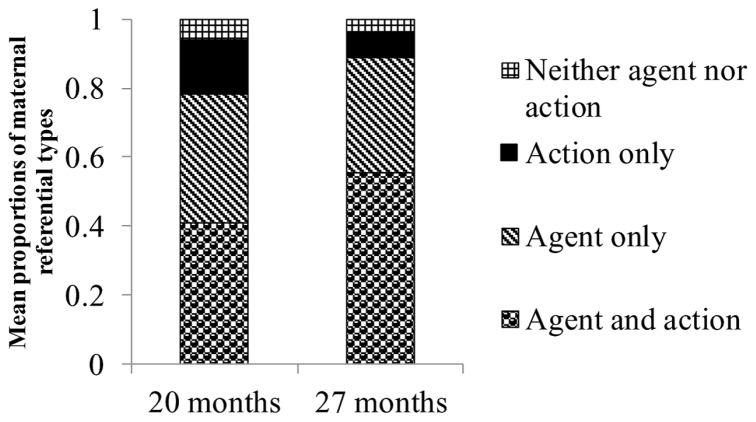
**Mean proportions of mothers’ and children’s referential types for information-giving and information-seeking utterances about a picture**.

Mothers changed their referential style from information giving to information seeking as their children got older. Moreover, mothers transferred the role of information giving to their children as the children grew older. We included information giving by mothers, information seeking by mothers, and information giving by children in our analyses of the referential style with which mothers and children responded to each picture. A series of ANCOVAs on the proportions of the four categories (agents and actions, agents only, actions only, and neither agents nor actions) again revealed a main effect of children’s age on actions-only responses, *F*(1,16) = 6.28, *p* < 0.05, η^2^ = 0.28. The proportion of references to only actions decreased between 20 and 27 months of age (*M* = 0.16, SD = 0.15 at 20 months; *M* = 0.08, SD = 0.11 at 27 months). Additionally, we also found a marginal main effect of children’s age on responses that included references to both agents and actions, *F*(1,16) = 3.48, *p* < 0.10, η^2^ = 0.18. The proportion of references to agents and actions increased between 20 and 27 months of age (*M* = 0.41, SD = 0.22 at 20 months; *M* = 0.55, SD = 0.22 at 27 months, **Figure 5**). When we used the productive vocabulary size reported by mothers at 20 months, the ANCOVA on references to agents and actions revealed no main effect of children’s age.

These results show mothers decreased actions-only references and increased agents-and-actions references as their children got older, although the results for agents-and-actions references are not robust. The results show that mothers did not significantly change the proportion of agents-only references.

### EFFECTS OF MATERNAL CONVERSATIONAL STYLES WHEN CHILDREN WERE 20 MONTHS OF AGE ON CHILDREN’S PRODUCTIVE VOCABULARY SIZE AT 27 MONTHS

We first examined the zero-order correlations between maternal conversational styles when children were 20 months of age and children’s observed productive vocabulary size at 27 months. We then partialed out the factor of children’s observed productive vocabulary size at 20 months and examined the association between maternal conversational styles when their children were 20 months of age and children’s observed productive vocabulary size at 27 months. We examined maternal conversational styles in terms of initiation styles (proportions of conversations about agents and actions initiated by maternal information seeking and information giving), response styles (proportions of maternal utterances involving elaborative information giving, elaborative information seeking, and other-repetition in response to children’s provision of information about agents and actions), and referential styles (proportions of references to both agents and actions, to agents only, and to actions only). These associations are presented in **Table 3**. We found a significant positive zero-order relationship between maternal elaborative information-seeking responses to children’s provision of information about agents at 20 months of age and children’s observed productive vocabulary size at 27 months [*r*(12) = 0.61, *p* < 0.05]; this association was marginally significant even when we controlled for children’s observed productive vocabulary size at 20 months [*r*(11) = 0.53,* p* < 0.10].

**Table 3:** Zero-order and partial correlations between maternal conversational styles when children were 20 months of age and children’s observed productive vocabulary size at 27 months.

	Zero-order correlation	Partial correlation
**Maternal initiation of conversation about agents (*N* = 18)**
Information giving	-0.44^†^	-0.25
Information seeking	0.26	0.27
**Maternal initiation of conversation about actions (*N* = 18)**
Information giving	-0.53*	-0.35
Information seeking	0.58*	0.41
**Maternal response to children’s provision of information about agents (*N* = 14)**
Elaborative information giving	-0.23	-0.47
Elaborative information seeking	0.61*	0.53^†^
Other-repetition	-0.47^†^	-0.09
**Maternal response to children’s provision of information about actions (*N* = 10)**
Elaborative information giving	0.29	0.58
Elaborative information seeking	0.22	0.15
Other-repetition	-0.13	-0.16
**Maternal referential choice (*N* = 18)**
Agent and action	0.48*	0.33
Agent only	0.03	-0.21
Action only	-0.51*	-0.16

Children’s observed productive vocabulary size at 20 months was partialed out in the analysis of partial correlation. *p< 0.05, ^†^*p*< 0.10.

Finally, the proportion of maternal elaborative information-seeking responses to children’s provision of information about agents was entered into a hierarchical regression analysis, treating children’s observed productive vocabulary size at 27 months as the outcome variable. Children’s observed productive vocabulary size at 20 months was entered first, followed by the proportion of maternal elaborative information-seeking responses to children’s provision of information about agents at 20 months. The results of the hierarchical regression analysis are presented in **Table 4**. Maternal elaborative information-seeking responses to children’s provision of information about agents at 20 months was a marginally significant predictor of children’s observed productive vocabulary size at 27 months. No other maternal conversational style when children were 20 months of age was a significant predictor when entered in the second step. We also performed a hierarchical regression analysis, treating children’s reported productive vocabulary size at 27 months as the outcome variable. We first entered children’s reported productive vocabulary size at 20 months as the control factor and then entered the proportion of maternal elaborative information-seeking responses. The analysis indicated that maternal elaborative information seeking was not a significant predictor of children’s productive vocabulary size at 27 months.

**Table 4:** Hierarchical regression analysis on the ability of maternal elaborative information-seeking responses to children’s provision of information about agents at 20 months to predict children’s observed productive vocabulary size at 27 months (*N* = 14).

	Model 1			Model 2		
	*B*	**SE* B***	β	*** B***	**SE* B***	β
Children’s productive vocabulary size at 20 months	1.23	0.35	0.71^**^	0.97	0.34	0.56^*^
Maternal elaborative information-seeking at 20 months				71.00	34.35	0.40^†^
*R*^2^		0.51			0.65	
*F* for change in *R*^2^		12.34^**^			4.27^†^	

* *p* < 0.05, **p < 0.01, ^†^*p* <0.10.

## DISCUSSION

The present study revealed that mothers increased the degrees of freedom available to by their children as a function of their children’s age in two ways. First, mothers broadened the range of their references by reducing the proportion of references to actions only and increasing the proportion to both agents and actions as their children aged. Second, mothers increased the proportion of conversations about actions that they initiated with information-seeking utterances as the age of their children increased. Maternal elaborative information-seeking responses to children’s provision of information include utterances that broadened the range of reference and required that children provide information. Although the proportion of maternal elaborative information-seeking responses to children’s utterances providing information about agents did not significantly increase as children aged, the proportion of maternal elaborative information-seeking utterances was moderately positively related to children’s productive vocabulary size at both 20 and 27 months. These results indicate that mothers introduce more complex information into their conversations with their children and transfer the role of actively producing information during conversations to their children as their children develop.

Maternal elaborative information-seeking responses to children’s provision of information about agents at 20 months was positively associated with children’s observed productive vocabulary size at 27 months. This result suggests that maternal responses that elaborate on information provided by their children and require children to adopt an active role in producing information promote the development of children’s vocabularies during the period from the one-word to the syntactic stage.

In addition to the aforementioned increase in the degrees of freedom, the present study revealed that mothers reduced the proportion of utterances designed to maintain their children’s attention and provide models, the other aspects of scaffolding. Mothers reduced other-repetition in their responses to children’s provision of information about agents as children developed. We discuss the individual maternal conversational styles in greater detail in the following sections.

### INITIATION OF CONVERSATION

In terms of conversations about actions, mothers reduced initiation by information giving and increased initiation by information seeking from 20 to 27 months, as proposed by Hypothesis 1. Contrary to Hypothesis 1, mothers did not initiate a significantly larger proportion of conversations about agents by information seeking, although they did initiate a smaller proportion of such conversations by information giving during this interval.

The absence of an increase in the proportion of conversations about agents initiated by information seeking can be explained by the age range of the children in this study. The mothers of the 20-month-old children included in the present study initiated conversations about agents in more than 50% of the episodes on average, which exceeded the proportion of conversations about actions initiated by information seeking by mothers of 27-month-old children. Previous studies have shown that the proportion of conversations initiated by maternal information-seeking utterances while reading picture books about objects substantially increased during the first half of the second year of their children’s life; however, the rate at which such utterances increased was reduced during the second half of the children’s second year (DeLoache and DeMendoza, 1987; Murase et al., 1998). The mothers of 20-month-old children included in the present study reached the level at which they frequently initiated conversations about agents with information-seeking utterances.

Why do mothers frequently initiate conversations about agents by information seeking when their children are 20 months of age? The first reason for this is the earlier acquisition of words about agents than about actions. Children produce utterances about agents more frequently than about actions starting at 20 months of age. Another reason for this finding is the order of conversations. In general, conversations about agents precede those about actions. At the beginning of such conversations, mothers are likely to use rhetorical questions that take the form of information-seeking utterances in the absence of an expectation of a reply from the addressee (DeLoache and DeMendoza, 1987). When mothers start a conversational episode about a picture, they may use rhetorical questions for the purpose of attracting children’s attention irrespective of whether the mothers expect an answer.

In summary, mothers shift their approach to initiating conversations from information giving to information seeking during the second year of their children’s lives. This shift occurs first in conversations about agents and next in conversations about actions. It represents an increase in the degrees of freedom in the scaffolding process that are controlled by children (Wood et al., 1976).

### RESPONSES TO CHILDREN’S INFORMATION-GIVING UTTERANCES

As hypothesized (hypothesis 2) mothers decreased other-repetition responses to children’s information-giving utterances about agents as their children aged from 20 to 27 months. This result is consistent with previous research (Murase et al., 1998). In general, previously established referents are more likely to be expressed in non-lexical (null or pronominal) forms than are newly introduced referents (Guerriero et al., 2006). In contrast to this principle, mothers of younger children may repeat referents that have been previously expressed by their children in an idealized form with the expectations that the children will imitate them and that the repetition will maintain their children’s focus on the ongoing conversation. The results show that mothers reduced these types of scaffolding (i.e., demonstration and direction-maintenance) as their children aged.

However, contrary to hypothesis 2, mothers did not significantly change the proportion of elaborative information-seeking and elaborative information-giving utterances in response to children’s information-giving utterances about agents as their children developed from 20 to 27 months. Elaborative information seeking can be viewed as providing less scaffolding and raising the ante because it adds new information to that provided by children and it asks children to produce information. Why did elaborative information-seeking responses not increase with children’s age? Mothers may decide whether they require elaborative information from their children based on their children’s ability to produce lexical utterances because maternal elaborative information-seeking responses are requests made after children provide information; this differs from requests for information that initiate conversations. In effect, the present study found a moderately positive relationship between the proportion of maternal elaborative information-seeking responses and children’s productive vocabulary size. This finding is in accord with the fact that the increase in the elaborative information-seeking utterances by Japanese mothers in response to their children’s labeling is more strongly related to their children’s productive vocabulary size than to their general developmental level, as represented by their children’s age (Murase et al., 2005).

Mothers’ responses to information-giving utterances about actions made by children did not change with children’s age. One reason that no such change was found could be the small number of information-giving utterances about actions produced by children. More data are required to reach conclusions about mothers’ responses to this kind of utterance. Another reason for the absence of an association between these utterances and children’s age involves the order in which the conversations proceeded. In response to many of the pictures, mothers and children started conversations by referring to the agents, and their references to actions came later. Mothers’ responses to children’s information-giving utterances about actions may have been affected by the preceding conversations about agents, which may have masked changes in mothers’ responses as children got older.

### MOTHERS’ REFERENTIAL CHOICES

Mothers broadened the range of their references as their children developed from 20 to 27 months. As hypothesis 3 proposed, the proportion of references to actions only decreased with children’s age. However, the proportion of references to both agents and actions did not significantly increase with children’s age when we limited the analysis to mothers’ information-giving utterances. When we considered the shift from providing to requesting information and the passing of the role of providing information from mother to child, we added information-seeking utterances by mothers and information-giving utterances by children to the category. We found that the proportion of references to agents and actions tended to increase as the children developed from 20 to 27 months, although this result was not robust.

Contrary to hypothesis 3, the proportion of references to agents only did not change with children’s age. This can be explained by the dominance of agentic references in this situation. Japanese mothers are more oriented to nouns/naming than to verbs/activity during mother–child conversations while reading picture books (Ogura et al., 2006). When their children are 20 months of age, mothers refer to things that can interest or be comprehended or produced by their children irrespective of whether the referents are agents or actions. When children reach 27 months of age, mothers focus on agentic references, on the one hand, while broadening the range of references, on the other.

In summary, mothers tend to broaden the range of references as their children grow older, which can be viewed as raising the ante because mothers appear to introduce more complex conversations to their children. This can also be viewed as reducing the degree of scaffolding because mothers tend to encourage their older children to be in control of a wider range of information. However, mothers also shift their focus toward agentic references as their children develop, and the decrease in references to agents only may in part reflect this shift.

### CONTRIBUTION OF MATERNAL ELABORATIVE INFORMATION-SEEKING UTTERANCES TO CHILDREN’S VOCABULARY DEVELOPMENT

The hierarchical regression analysis showed that maternal elaborative information-seeking responses to children’s information-giving utterances about agents at 20 months of age marginally predicted increases in children’s observed productive vocabulary size. Although not robust, this result suggests that maternal conversational style can contribute to children’s language development as children transition from the one-word to the syntactic stage.

Maternal elaborative information-seeking utterances have three important characteristics. First, they extend the scope of conversations in that mothers use them to add new information. The results of the present study are consistent with findings that maternal lexical richness, which is exemplified by elaborative information-seeking utterances, exerts a positive effect on children’s vocabulary development (Bornstein et al., 1998; Hoff and Naigles, 2002). Second, maternal elaborative information-seeking utterances act as requests for children to take an active role in the conversation by providing information. The results of the present study are consistent with findings that dialogic reading is positively related to children’s language development in that it includes asking wh-questions and following children’s answers with questions (Arnold and Whitehurst, 1994). Third, maternal elaborative information-seeking utterances are responsive in that they are contingent on and appropriately connected to children’s utterances. The results of the present study are in accord with earlier research indicating that maternal responsiveness contributes to children’s vocabulary development (Tamis-LeMonda et al., 2001; Tamis-LeMonda and Bornstein, 2002).

In summary, adding new information, requesting information, and being responsive may contribute to children’s vocabulary development during the transition from the one-word to the syntactic stage.

### LIMITATIONS AND FUTURE DIRECTIONS

The conclusions drawn from this study and their generalizability are limited by some aspects of the design. Although the 7-min observation period was substantial, only a modest number of information-giving utterances were provided by some children. Thus, the power of this research to analyze maternal responses to children’s provision of information was limited. Additionally, we did not analyze information about arguments because too few relevant utterances were made.

The results regarding increased references to both agents and actions and the contribution of maternal elaborative information-seeking utterances to children’s vocabulary development are not robust. Additional research is needed to confirm these results.

Conversations during reading other types of books (e.g., story books) need to be investigated because the types of utterances made by mothers change according to the kinds of picture that is viewed (Chan et al., 2009). It would also be interesting to examine conversations that occur while mothers and children are viewing animated scenes. Indeed, unlike the static scenes used in this study, animated scenes may elicit more utterances about actions from mothers and children during joint watching. Furthermore, conversations during joint picture-book reading are more focused on labeling than are conversations in other situations, such as playing with toys. It would be important to investigate whether mothers modify their utterances in other situations in a way that is similar to the patterns observed in the present study.

Japanese is a pro-drop language, and we need to investigate whether our findings about referential choice can be generalized to the speakers of other languages. For example, in English, the agents, actions, objects, and recipients are all explicitly mentioned. We need to examine whether English-speaking caregivers change the range of their references as a function of children’s age.

Another interesting topic for future study would be cross-cultural comparisons between East Asian and North American caregivers. Although information about differences in the attentional styles of East Asians and North Americans has been increasing (Masuda and Nisbett, 2001; Masuda et al., 2008), only a few studies have attempted to clarify the developmental processes underpinning cross-cultural differences (Chan et al., 2009). Thus, the comparisons of the referential choices made by Japanese and American caregivers need to be investigated from a cross-cultural perspective.

## CONCLUSION

This study determined that Japanese mothers decrease scaffolding and raise the ante by changing the balance between providing and requesting information and by broadening their range of references. This study also suggests that maternal elaborative information-seeking responses contribute to children’s vocabulary development. Cross-cultural studies will clarify the universality and culture-specific aspects of these characteristics and elucidate the developmental processes underlying children’s language development.

## Conflict of Interest Statement

The author declares that the research was conducted in the absence of any commercial or financial relationships that could be construed as a potential conflict of interest.
